# Spotted hyena optimizer algorithm for capacitor allocation in radial distribution system with distributed generation and microgrid operation considering different load types

**DOI:** 10.1038/s41598-021-82440-9

**Published:** 2021-02-01

**Authors:** Amirreza Naderipour, Zulkurnain Abdul-Malek, Mohammad Hajivand, Zahra Mirzaei Seifabad, Mohammad Ali Farsi, Saber Arabi Nowdeh, Iraj Faraji Davoudkhani

**Affiliations:** 1 Institute of High Voltage and High Current, School of Electrical Engineering, Faculty of Engineering, Universiti Teknologi Malaysia, 81310 Johor Bahru, Malaysia; 2 Young Researchers and Elite Club, Borujerd Branch, Islamic Azad University, Borujerd, Iran; 3 Aerospace Research Institute, Ministry of Science, Research and Technology, Tehran, Iran; 4 Science and Research, Tehran Branch, Islamic Azad University, Tehran, Iran; 5 Golestan Technical and Vocational Training Center, Gorgan, Iran; 6 Department of Electrical Engineering, Islamic Azad University, Khalkhal Branch, Khalkhal, Iran

**Keywords:** Electrical and electronic engineering, Energy science and technology

## Abstract

In this paper, the optimal allocation of constant and switchable capacitors is presented simultaneously in two operation modes, grid-connected and islanded, for a microgrid. Different load levels are considered by employing non-dispatchable distributed generations. The objective function includes minimising the energy losses cost, the cost of peak power losses, and the cost of the capacitor. The optimization problem is solved using the spotted hyena optimizer (SHO) algorithm to determine the optimal size and location of capacitors, considering different loading levels and the two operation modes. In this study, a three-level load and various types of loads, including constant power, constant current, and constant impedance are considered. The proposed method is implemented on a 24-bus radial distribution network. To evaluate the performance of the SHO, the results are compared with GWO and the genetic algorithm (GA). The simulation results demonstrate the superior performance of the SHO in reducing the cost of losses and improving the voltage profile during injection and non-injection of reactive power by distributed generations in two operation modes. The total cost and net saving values for DGs only with the capability of active power injection is achieved 105,780 $ and 100,560.54 $, respectively and for DGs with the capability of active and reactive power injection is obtained 89,568 $ and 76,850.46 $, respectively using the SHO. The proposed method has achieved more annual net savings due to the lower cost of losses than other optimization methods.

## Introduction

Application of distributed generations (DGs) in distribution systems due to growth in energy demand has many advantages such as reducing power losses and system cost, improving the voltage profile and power quality, and improving reliability^1^. Different definitions for distributed generations are presented, but the comprehensive definition, without limitation, is "the source of electrical energy directly connected to the distribution network or consumer side"^2^. The application of DGs in distribution networks has become an important topic in recent years and has influenced the operation and design of these networks^3^. Installation of DGs in non-optimal locations increases loss, weakens the voltage profile, and increases distribution costs for network users, contrary to expectations^4^. Therefore, DG resources should be optimally allocated in the distribution network to maximise the benefits^5^. Studies show that approximately 13% of the power generated in the system is wasted as Ohm’s losses in the distribution network^6^. In the operation of distribution networks, various methods have been used to improve the network characteristics especially reduction of losses. Also, capacitors in the distribution networks are widely used for reactive power compensation because of their low cost, low losses, minimal need for repair and maintenance, and higher economy than other methods, including reactive power compensators^7^. Unsuitable capacitor placement in networks that use capacitors increases the losses and the cost of generating and transmitting energy^7^. Therefore, it is necessary to optimise the location and size of capacitors in the distribution network. A microgrid is a set of loads, DGs, and in some cases generating equipment that operates as a controllable load or generator that can provide power and heat for a local area and operate in either islanding or grid-connected modes^8^. The reactive power compensation based on optimal capacitors placement in islanding and grid-connected modes in microgrids operation can be one of the attractive fields of distribution network operators^9^. In recent years, the use of meta-heuristic optimization methods to determine the optimal location and size of reactive sources has been welcomed due to their high efficiency in achieving optimal results. Therefore, optimization methods with different capabilities in terms of convergence power and accuracy are presented.

Several studies have been conducted on optimal capacitor placement in distribution networks, with different objective functions and different optimization methods. In^10^, capacitor placement is presented by the teaching learning-based optimization (TLBO) method to determine the size of all reactive power sources and is implemented to minimize cost and power losses by considering a constant and effective load model using the modified TLBO (MTLBO) method. In^11^, multi-objective optimization of locating distributed generation resources and capacitors in the distribution network is presented using modified particle swarm optimization (MPSO) algorithm to reduce losses and improve the voltage stability and load balancing index. In^12^, the optimal location of distributed generation resources and capacitors in the distribution network is determined using the imperialistic competitive algorithm (ICA) and genetic algorithm (GA) to reduce losses, improve the voltage profile, increase the voltage stability index, and balance the load. In^13^, a cuckoo search algorithm (CSA) is proposed for optimal placement of shunt static capacitors in distribution networks to minimize the cost of reactive power in different loading conditions and improve the voltage profile. In^14^, long-term planning was performed to optimally allocate capacitors in radial distribution systems to minimize power losses using the bacterial foraging optimization (BFO) algorithm. In^15^, optimal placement and sizing of capacitors in the distribution network are presented to reduce losses, improve voltage stability, and reduce the cost of purchasing and installing capacitors using the artificial bee colony (ABC) algorithm. In^16^, the location of a capacitor in a radial distribution network is presented using the PSO algorithm to reduce the costs of energy losses and of installing and purchasing capacitors. In^17^, the optimal location of the capacitor and the optimal reconfiguration of the distribution network to reduce distribution network losses by considering different load models is presented using the shrimp straw algorithm. In^18^, the flower pollination algorithm (FPA) is used to locate and determine the optimal sizing of capacitive banks in the distribution network. In^19^, the biogeography-based optimization (BBO) method is used to determine the optimal sizing of capacitive banks in a distribution network to reduce the costs of active and reactive power losses and the energy purchased from the upstream network. In^20^, the optimal location of capacitors to reduce losses and costs is presented using the harmony search algorithm (HSA). In^21^, the optimal location and sizing of capacitors in a distribution network using the bacterial foraging algorithm (BFO) to reduce power loss is presented. In^22^, the optimal placement and sizing of parallel capacitors in the distribution network are optimally determined to minimize losses and maximize financial gain due to the use of capacitors using gravitational search slgorithm (GSA). In^23^, flower pollination algorithm (FPA) is applied for solving the capacitor placement problem in the network with aim of total power loss and capacitor installation cost minimization. In^24^, allocation of capacitors is studied via the sine–cosine algorithm (SCA) for maximizing the net saving and reliability enhancement. In^25^, modified cultural algorithm (MCA) is used to reduce the power loss of the distribution networks by capacitor allocation. In^14^, allocation of capacitors in the networks aimed with minimizing the power loss via bacterial foraging optimization algorithm (BFOA). In^26^, optimal location and sizing of the capacitor are studied to minimize the power losses and enhancement of voltage profile via cuckoo search algorithm (CSA). In^27^, allocation of DGs and shunt capacitor in the distribution networks are presented to reduce the power losses using artificial bee colony (ABC) algorithm. In^28^, autonomous group particle swarm optimization (AGPSO) is applied for optimal allocation and sizing of DGs and capacitors for minimizing the power losses. In^29^, the thief and police optimization algorithm (TPOA) is developed simultaneous network reconfiguration integrated with the allocation of the capacitor and renewable energy resources with minimizing the power loss and cost as well as voltage stability enhancement. In^30^, hybrid GA-PSO algorithm is presented to solve the allocation problem of DGs for enhancing the active and reactive loss and also voltage regulation. In^31^, allocation of the DGs and capacitors in the distribution networks is presented to reduce the losses and voltage deviations and also to improve the voltage stability via PSO. In^32^, water cycle algorithm (WCA) is developed to allocate the DGs and capacitors in the radial networks with minimizing the losses, voltage deviation, energy cost, emissions and also voltage stability enhancement. In^33^, an enhanced genetic algorithm (EGA) is applied for allocation of DGs and capacitors in the networks with losses and voltage deviation minimization.

Investigating the literature review cleared that the meta-heuristic method is very effective in determining the best location and capacity of reactive resources as well as distributed generation resources to achieve the best performance of the distribution network. Therefore, it is necessary to use the meta-heuristic methods with high optimization power and also the low computational cost to achieve the best network performance. One of the most powerful optimization methods that have been presented recently is called the spotted hyena optimizer (SHO) based swarm‐based algorithm. The SHO is inspired by hunting and social behaviours of the spotted hyenas for solving realistic engineering problem with constraint and unconstrained challenges. In^34^, the SHO is suggested to solve two real-life design problems named optical buffer and airfoil design. The obtained results showed that the SHO is an effective optimizer method for solving these problems and produce near-optimal designs. In^35^, the SHO is presented for solving matching of a complication image problem. The results cleared that the SHO is more superior in the matching of complication image than the PSO, ABC, ICA and GWO. In^36^, the SHO is developed to solve convex and non-convex economic dispatch. The optimization results showed that the SHO is capable to solve the economic dispatch problems and this method can converge to global optimal with lower computational cost and better performance of the SHO in problem solution is proved compared with CSA and BBO. In^37^, deterministic and probabilistic allocation of wind energy resources in the distribution networks is presented with the aim of losses and voltage deviation reduction and also voltage stability enhancement using the SHO. The superiority of the suggested method is proved given better losses, voltage profile and stability with faster convergence speed and lower computational cost compared with PSO. In^38^, the SHO is applied for travelling salesman problems. The results indicated that the SHO provided the optimal solutions for travelling salesman problems and its superiority is confirmed compared with well-known GA, ACO, and PSO algorithms. In^39^, the SHO is developed for optimization of proportional integral derivative (PID) parameter in automatic voltage regulator (AVR) system. The obtained results showed the better performance of the SHO than the PSO, SCA, FPA and GWO algorithms given better convergence speed and accuracy. In^40^, the optimal power flow in microgrid based on renewable energy resources is presented using the SHO. The SHO is applied to the optimal determination of controller parameters due to voltage variations. The result cleared that the method based SHO is more effective than the other results especially the PSO. In^41^, the allocation of DGs with network reconfiguration is implemented in the distribution networks aimed with minimizing the losses and voltage deviations and the achieved results are compared with PSO and DE methods which the effectiveness of the SHO is confirmed. In^42^, the SHO is applied to resolve complicated nonlinear physical world tasks and the superiority of the SHO is confirmed with explorative strength compared to the other algorithms. In^43^, as can be seen from recent studies on the use of the SHO method to solve the optimization problems, it is a meta-heuristic algorithm with high power and rate of convergence compared with well-known algorithms. Moreover, the comparisons between the SHO and of other meta-heuristic methods showed that the SHO can handle different types of constraints and offer better solutions than the other optimizers. So, these advantages are main reasons for using the SHO to solve the optimization problem in this study. Moreover, the focus of capacitor allocation in a literature review is based on the objective function type and the use of new optimization methods. The literature review showed that the problem of capacitors placement and sizing along with the optimal allocation of distributed generation resources with the capability to be exploited as microgrids due to islanding is not well addressed in the literature review. In most studies, the effect of locating these two devices has been done integrated with the whole network. Also, in the actual operation of the network, it is better to consider the effect of load changes as a light load, normal load and heavy load periods and to avoid considering the load as a constant. It is also more desirable to evaluate the effect of different types of load including constant power load, constant current load and also constant impedance load. A review of previous studies has shown that the effect of changes in load levels as well as load types together is not well addressed well.

In this paper, the optimal allocation of capacitors in distribution networks is presented to minimize the cost using spotted hyena optimizer (SHO)^44^ considering different levels and types of loads. The proposed method is implemented on the 24-bus network that the part of the network is disconnected from the network due to islanding state, which is supplied in the form of microgrids using the distributed generation resources. The system cost is considered as minimization of the energy losses cost, power losses cost in peak load conditions as well as installation, purchase and maintenance costs of the capacitor. DGs utilization is incorporated in two cases including with only active power injection and active and reactive power injection. In this study, three levels load including light, normal, and heavy loads and also different load types as constant power, constant current, and constant impedance are selected in the problem solution for the realistic operation of the network. The performance of the SHO in capacitor allocation is evaluated in two grid-connected and microgrid modes compared with the well-known GWO algorithm.

The highlights of the paper are presented as follows:

- Optimal allocation of capacitors in distribution networks with the presence of distributed generations
- Multi-criteria objective function as minimizing the costs of energy losses cost, power losses, and capacitor application
- Distribution network operation in grid-connected and microgrid (islanding) modes
- Problem solution incorporating three levels of the load and also different load types
- Using of spotted hyena optimizer (SHO) for optimal allocation of capacitors in distribution networks
- The superiority of the SHO compared to well-known GWO algorithm

In “Problem formulation” section of this paper, the problem formulation, including load modelling, objective function, and optimization problem constraints, is presented. In “Proposed optimization method” section, the proposed optimization method and its implementation in problem solution are described. The simulation results are presented in “Simulation results and discussion” section, and the conclusion is presented in “Conclusion” section.

## Problem formulation

In this section, the formulation of the optimal capacitor placement problem in DG-embedded distribution networks is presented. Also, the objective function and problem constraints are described.

### Load model

In this study, three types of load are considered. The mathematical representation of active and reactive power loads is as follows^45^:

1 $${{P}_{L}={P}_{L0}({a}_{1}+a}_{2}\left|V\right|+{a}_{3}{\left|V\right|}^{2})$$

2 $${{Q}_{L}={Q}_{L0}({b}_{1}+b}_{2}\left|V\right|+{b}_{3}{\left|V\right|}^{2})$$

where (*a*_*1*_*, b*_*1*_), (*a*_*2*_*, b*_*2*_), and (*a*_*3*_*, b*_*3*_) are the components of constant power, constant current, and constant impedance, respectively. For a constant power load, *a*_*1*_ = *b*_*1*_ = 1 and *a*_*2*_ = *b*_*2*_ = *a*_*3*_ = *b*_*3*_ = 0; for a constant current load, *a*_*2*_ = *b*_*2*_ = 1 and *a*_*1*_ = *b*_*1*_ = *a*_*3*_ = *b*_*3*_ = 0; and for a constant impedance load, *a*_*3*_ = *b*_*3*_ = 1 and *a*_*1*_ = *b*_*1*_ = *a*_*2*_ = *b*_*2*_ = 0.

### Objective function

The objective function in this study is the cost, which has three terms. The first term is the cost of energy losses. The second term is the cost of losses in heavy loading condition, and the third term is the cost of installation and maintenance of the capacitor. This objective function can be represented as follows.

3 $${objFun=CE}_{loss}+{CP}_{loss}^{peak}+{C}_{capacitor}$$

### Energy losses cost

This section contains the total daily losses of energy at all three levels of loading and in two network performances or two scenarios i.e. connected to grid and islanding mode, which is shown below.

4 $${CE}_{loss}={C}_{e }\left({\sum }_{j=1}^{M}{\sum }_{i=1}^{{N}_{GC}-1}{P}_{loss}^{j,GC}\right)\left({\Delta \mathrm{T}}_{j }-{P}_{i,j }{\Delta \mathrm{T}}_{j }\right)+({\sum }_{j=1}^{M}{\sum }_{i=1}^{{N}_{Is}-1}{P}_{{loss}_{i }}^{j,Is})({P}_{i,j }{\Delta \mathrm{T}}_{j })$$

where, C_e_ is energy cost ($/kWh), M is the number of load levels, N_GC_ and N_Is_ are the number of buses in grid-connected and islanded modes respectively, $${P}_{loss}^{j,GC}$$ and $${P}_{{loss}_{i }}^{j,Is}$$ are the ith line losses in jth load level in grid-connected and islanded modes respectively (kW), $${\Delta \mathrm{T}}_{j}$$ is the period of load level *j* for 1 year (h) and $${P}_{i,j}$$ is the probability of operation in islanded mode at the load level j.

### Losses cost in peak load conditions

In peak conditions, the capacity of the lines is close to their maximum value and most of the equipment works at its maximum capacity. Hence, reducing peak losses can be very beneficial causing some parts of the equipment capacity to be empty and increase their lifespan. This section can be expressed as follows.

5 $${CP}_{loss}^{peak}={C}_{p }[\left({\sum }_{i=1}^{{N}_{GC}-1}{P}_{{loss}_{i }}^{peak,GC}\right)+\left({\sum }_{i=1}^{{N}_{Is}-1}{P}_{{loss}_{i }}^{peak,Is}\right)]$$

where the annual cost of energy is at the load peak ($/kWh-year), $${P}_{{loss}_{i }}^{peak,GC}$$ and $${P}_{{loss}_{i }}^{peak,Is}$$ are the number of losses at heavy load level in a network-connected and islanded mode.

### Capacitor cost

In this study, both fixed and switchable capacitors are applied. These two types of capacitors include installation, purchase, and maintenance costs. The total cost of the capacitors is formulated as below:

6 $${C}_{capacitor}={(M}_{f }.{C}_{if }+{\sum }_{i=1}^{{N}_{GC}}{C}_{vf }.{Q}_{fi })+({M}_{s }.{C}_{is }+{\sum }_{i=1}^{{N}_{GC}}{C}_{vs }.{Q}_{si })$$

where *M*_*f*_ and *M*_*s*_ are the numbers of constant and switchable capacitors, $${C}_{if}$$ and $${C}_{is}$$ are the cost of purchasing and installing of constant and switchable capacitors ($),$${C}_{vf}$$ and $${C}_{vs}$$ are the repair and maintenance cost of constant and switchable capacitors ($/kVAR-year), $${Q}_{fi}$$ and $${Q}_{si}$$ refer to the amount of constant and switchable reactive power (kVAR) respectively.

### Problem constraints

The capacitor placement problem must be optimized subject to operational constraints. The most important constraints regarding this problem are the voltage magnitude of buses, transmitted power bylines, the amount of reactive power installed on the network and capacitor installation rates according to the available budget. These four constraints are listed below:

7 $${V_{i,min}} \leqslant {V_i} \leqslant {V_{i,max}}$$

8 $${I_i} \leqslant {I_{i,max}}$$

9 $$\mathop \sum \limits_{i = 1}^{\text{N}} Q_{capacito{r_{i\;}}}^j \leqslant k.\mathop \sum \limits_{i = 1}^{\text{N}} Q_{Loa{d_{i\;}}}^j$$

10 $${C_{capacitor}} \leqslant {\text{AB}}$$

where ${V}_{i}$, ${V}_{i,min}$ and ${V}_{i,max}$ are the voltage of bus *i*, the minimum and maximum of allowed voltages, ${I}_{i}$ and ${I}_{i,max}$ are line current and the thermal limit of line *i*, ${Q}_{{capacitor}_{i }}^{j}$ and ${Q}_{{Load}_{i }}^{j}$ are the installed reactive power and consumed in the bus *i* and the load level *j* respectively, *N* refers to the number of buses, k represents the allowed percentage of installed reactive power, ${C}_{capacitor}$ is capacitor placement cost and AB is the total available budget of capacitor placement.

## Proposed optimization method

In this section, the overview of spotted hyena optimizer (SHO) algorithm and its implementation in problem solution are described.

### Overview of SHO

The behaviour of spotted hyena is sometimes similar to human social behaviour. They can fight tirelessly for food and life and usually live in tribes. Male hyena leaves their tribe when reaching puberty and join another tribe for an extended period. Interestingly, they use several sensory generators to recognise familiar people. Hyena informs each other by generating alarm sounds when reaching a new food source. The SHO method used in the present study is based on the hunting and social behaviours of these animals. The SHO can handle various types of constraints and offer better solutions than other powerful optimizers and also Does not get caught up in local optimal by increasing the number of problem variables as well as the complexity of the optimization problem and has high convergence speed and accuracy^44^.

### Encircling prey

Other searching factors can be considered their best position related to the prey or target as the best response and update it. The mathematical model of this behaviour is expressed as follows^44^:

11 $${D}_{h}=\left|B.{P}_{p}\left(x\right)-P\left(x\right)\right|,$$

12 $$P\left(x+1\right)={P}_{p}\left(x\right)-E.{D}_{h},$$

where *D*_*h*_ represents the distance between the spotted hyena and the prey, *P*_*P*_ represents the position vector related to the prey, *P* represents the position vector of the spotted hyena, *X* represents the current iteration, and *B* and *E* represent the coefficient factor vectors^44^.

13 $$B=2.r{d}_{1},$$

14 $$E=2h.r{d}_{2}-h,$$

15 $$h=5-(\mathrm{Iteration }\times (5/{Max}_{\mathrm{Iteration }})),$$

Here,

16 $$\begin{aligned} {\text{Iteration}} & = 0,{\text{\;}}1,{\text{\;}}2, \ldots ,{\text{\;}}Ma{x_{{\text{Iteration\;}}}} \\ {\text{Iteration}} & = 0,{\text{\;}}1,{\text{\;}}2, \ldots ,{\text{\;}}Ma{x_{{\text{Iteration}},{\text{\;}}}} \\ \end{aligned}$$

where *rd*_*1*_ and *rd*_*2*_ are random vectors in the range of [0, 1], and *h* can be linearly reduced from 5 to 0.

### Hunting

The proposed SHO algorithm hunting strategy is described as follows^44^.

17 $${D}_{h}=\left|B.{P}_{h}-{P}_{k}\right|,$$

18 $${P}_{k}={P}_{h}-E.{D}_{h},$$

19 $${C}_{h}={P}_{k}+{P}_{k+1}+\dots +{P}_{k+N}$$

Here, *P*_*h*_ represents the best position of the spotted hyena related to the prey, and *P*_*k*_ represents another position of the spotted hyena. *N* represents the total number of spotted hyena and is calculated as follows.

20 $$N={count}_{nos}({P}_{h},{P}_{h+1},{P}_{h+2},\dots ,\left({P}_{h+M}\right))$$

Here, *M* represents a random vector in the range of [0.5, 1], *nos* represents the number of answers (the reference answers are counted), and ***C***_*h*_ represents a group of *N* optimal answers.

### Attacking prey (exploitation)

The mathematical formula for attacking prey can be defined as follows^44^.

21 $$P\left(x+1\right)=\frac{{C}_{h}}{N},$$

Here ***P ***(*x* + 1) saves the best pass and updates the positions of other factors relative to the position of the best search factor.

### Search for prey (exploration)

To identify the correct answer, ***E*** must be > 1 or < 1, according to Eq. (19). The other part of the SHO algorithm, which makes exploration possible, is ***B***. Vector ***B*** contains random values that provide the prey’s random weights according to Eq. (20). Suppose that the ***B*** > *1* vector has priority over the ***B*** < *1* vector to show more random behaviour of the SHO algorithm and the effect of the distance^44^.

### The SHO implementation

The steps of implementing the SHO in problem solution are presented:

- **Step 1.** Set optimization parameters such as population size, number of maximum iterations and repetition, number of variables (location and size of capacitors) and the variables constraint.
- **Step 2.** The initial population matrix is then generated. Each row represents a member of the population, and each column represents the bus number in which the capacitor is installed (The priority is to install the capacitor in the bus with higher loss sensitivity, which is detected by the optimization program).
- **Step 3.** Calculate the objective function (Total cost) considering the constraints per member Step 2 and the hyena with the lowest cost is selected as the representative in this step.
- **Step 4.** The SHO population is updated to achieve the next position using a continuous variable.
- **Step 5.** Round all members for moving to discrete search space to the nearest integer and also recalculate the objective function considering the constraints for all members. If the value of the new member's objective function is better than the value obtained in Step 3, replace with it.
- **Step 6.** In this step (termination criterion) if the number of iterations exceeds the maximum iterations number, the SHO will be stopped and otherwise it will return to step 4.

Finally, the results include the location and size of fixed and switchable capacitors, power loss and minimum network voltage at each load level in both islanding and grid-connected operation and the cost before and after capacitor installation are provided.

## Simulation results and discussion

**Figure 1:**
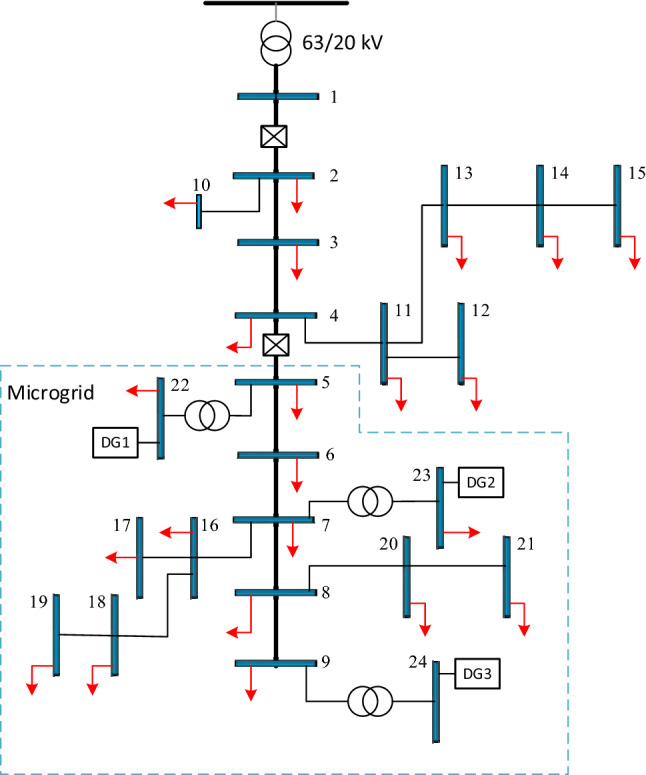
The single line diagram of 24-bus network^20^.

In this section, simulation results of optimal allocation of capacitors in distribution networks with non-dispatchable distributed generations are presented with operation capability as a microgrid to minimize cost using the gray wolf optimizer algorithm, considering the levels and types of load. The objective function of simulation is considered as minimization of energy losses costs, peak power losses and capacitor utilization and the proposed method is implemented on a 24-bus network. The basic objective of the capacitor allocation is power losses minimization in the distribution network. This objective determines the best locations for capacitors to be installed to obtain the minimum losses while the other places may not achieve this objective. This objective can be defined as the sensitivity of active power loss to reactive power injection at a bus. So the buses with higher sensitivities are selected as candidate bus for capacitor installation by the optimization program in this study. Further explanation of this is given in detail in Ref.^18^. The simulation results are presented in two operation modes of grid-connected and islanded (for microgrid), and the results before and after the optimization are investigated. Also, to verify the SHO method, the results were compared with GWO and also the results have been compared with the reference^45^ which the GA is employed to problem solution. The single-line diagram of the 24-bus network is shown in Fig. 1.

According to Fig. 1, a part of the 24-bus test case has distributed generation resources, which is called the microgrid section due to islanding state. The distributed generations are applied in two modes with unit power factor without the capability of reactive power injection and non-unit power factor with reactive power injection capability. Economic data of the network, distributed generations and its generation values and transformers, load and line data are presented in Tables 1, 2 and 3. Also, in this study, a light loading period is 1000 h with 2.175 MW and 1.875 MVAR, a normal loading period is 6760 h with 6.335 MW and 4.375 MVAR and heavy loading period with 1000 h, 9.05 MW and 6.25 MVAR during a year.

**Table 1:** The economic information of 24-bus network^20^.

Factor	Value	Unit	Factor	Value	Unit
B	25,000	$	C_p_	120	$/kW
P_Ij_	(0.0015, 0.00013 and 0.0006)	–	C_e_	0.1	$/(kW h-year)
T_j_∆	(1000, 6760, 1000)	h	C_if_	20	$
Q_SI,Max_	1.36	MVAr	C_vf_	3	$/(kVAr-year)
Q_SI,Min_	0	MVAr	C_is_	30	$
–	–	–	C_vs_	9	$/(kVAr-year)

**Table 2:** Information of DG resources, amount of production of distributed generation resources at different load levels^20^.

DG number	Rated power (MVA)	Rated power (MW)	Grid-connected mode			Islanding mode		
			Level 1	Level 2	Level 3	Level 1	Level 2	Level 3
DG_1_	1.41	1.20	1.2	1.2	1.20	1.2	1.20	1.20
DG_2_	2.35	2	0	2	2	0	2	2
DG_3_	2.59	2.2	1.65	2.2	2.2	Slack	Slack	Slack

**Table 3:** Transformers and load information^20^.

Sending bus	Bus number (ending bus)	P_L_ (p.u)	Q_L_ (p.u)	R (p.u)	X (p.u)	Rated power (MVA)
**Transformers**						
–	22	0.01	0.006	–	–	–
–	23	0.015	0.009	–	–	–
–	24	0.02	0.012	–	–	–
**Lines**						
5	22	–	–	0.0667	0.3	1.5
7	23	–	–	0.04	0.24	2.5
9	24	–	–	0.0267	0.2167	3

### Simulation results at cosφ = 1

**Figure 2:**
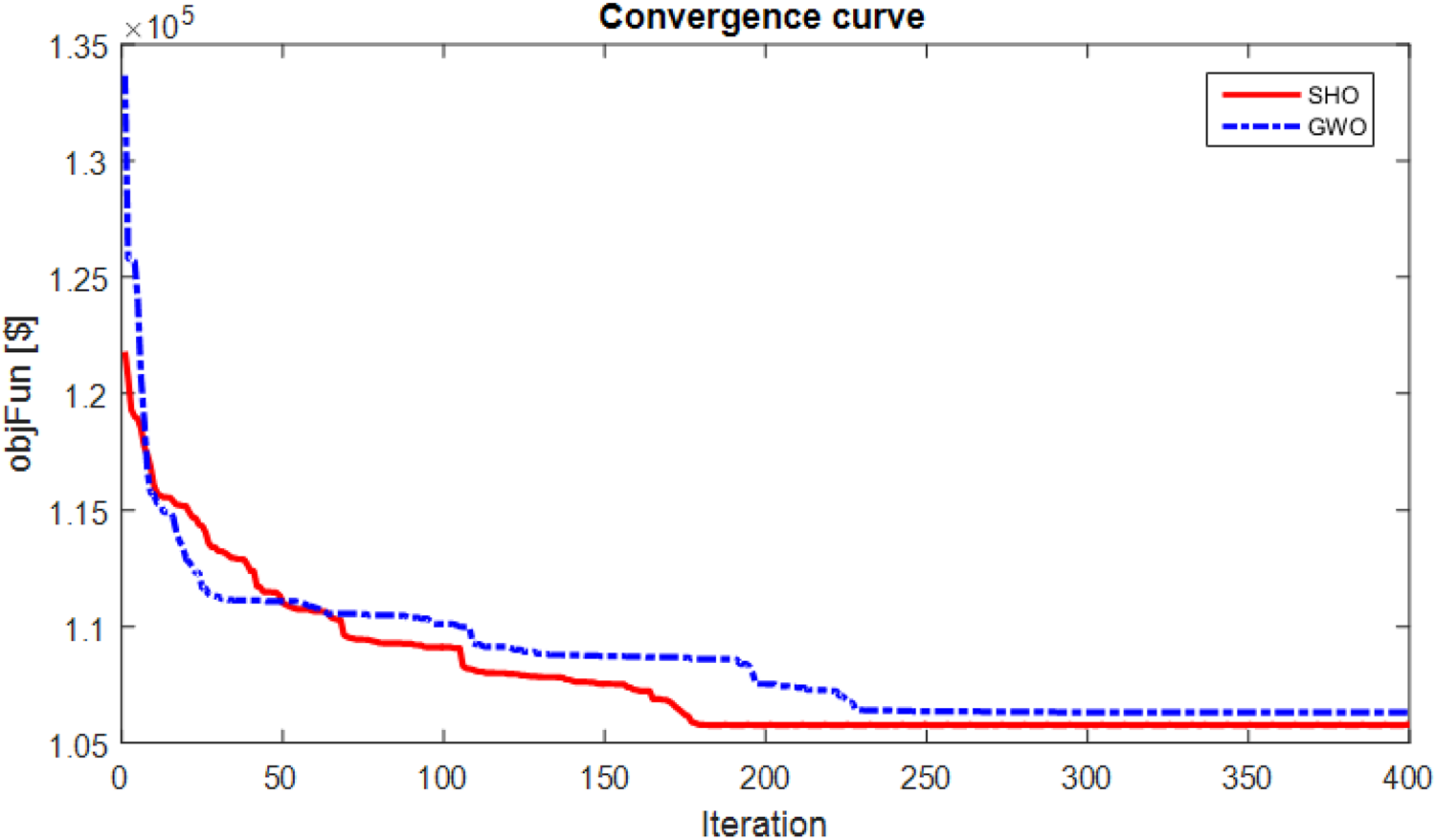
The convergence process of optimization methods for the 24-bus network with cosφ = 1.

In this section, it is assumed that distributed generation sources operate with a unit power factor and generate only active power. The convergence curve of SHO and GWO methods is presented in Fig. 2.

As shown in Fig. 2, the SHO method has a lower cost than the GWO for capacitor placement. Also, statistic analysis of different algorithms performance with cosφ = 1 due to 25 repetition is presented in Table 4. According to the obtained results, the SHO is achieved to lower-cost given Best, Mean, Worst and standard deviation (STD) values compared with the GWO.

**Table 4:** Statistic analysis of different algorithms performance with cosφ = 1.

Item/algorithm	SHO	GWO
Best ($)	105,780	106,310
Mean ($)	105,936	106,805
Worst ($)	106,492	107,570
STD ($)	4165	4858

The optimal location and sizing results of the capacitors by various methods of optimization in the 24-bus network are presented in Table 5. The SHO methods installed 2100 kVAR capacitors, at the second level (normal loading) and at the third level (heavy loading) too and also 1650 kVAR placed in normal and heavy loading by GWO. At the first level (light loading), SHO placed 1650 kVAR and GWO installed 1950 kVAR capacitor. In contrast, the GA method^20^ also installed 1650, 1500, and 2100 kVAR capacitors, at the first to third levels, respectively. So, the SHO has a value of 5850 kVAR, and GWO and GA methods each have a total of 5250 kVAR capacitor placement in the network. Various values of cost in the 24-bus network before and after the capacitor placement are given in Table 6. Before capacitor placement, the cost of network losses is 150,425.5 $ which after capacitor placement the amount of this cost in SHO, GWO, and GA^14^ methods is 59,447 $, 61,120 $, and 61,422.222 $, respectively. Total cost amount before capacitor placement is 206,340.54 $, which was reduced to 105,780, 106,310, and 107,228.51 after applying the capacitor by SHO, GWO and GA method^20^ respectively. Also, annual savings by SHO method are more than the other methods. Therefore, the SHO has better performance on reducing the cost of losses than the other methods.

**Table 5:** optimal capacitor placement on 24-bus network with cosφ = 1.

Bus number	Fixed capacitor			Switchable capacitor (Level 2)			Switchable capacitor (Level 3)		
	SHO	GWO	GA^20^	SHO	GWO	GA^20^	SHO	GWO	GA^20^
7	300	450	150	0	0	0	0	0	0
8	150	0	300	0	0	0	0	0	0
9	0	0	0	300	450	300	300	450	300
11	300	450	300	0	0	0	0	0	0
12	0	0	0	150	150	150	150	0	150
13	0	0	0	300	0	150	300	0	150
14	0	150	300	0	150	0	0	0	0
15	450	300	150	0	300	0	0	0	0
16	0	0	0	300	0	300	300	150	300
17	0	150	0	150	150	300	150	450	300
18	300	150	150	0	150	0	0	0	0
19	0	0	0	300	0	300	300	300	300
20	150	300	300	0	300	0	0	0	0
21	0	0	0	300	0	300	300	300	300
23	0	0	0	300	0	300	300	0	300
Total (kvar)	1650	1950	1650	2100	1650	1500	2100	1650	2100

**Table 6:** Different cost sections in the 24-bus network before and after the capacitor placement with cosφ = 1.

Parameter	Before cap placement	After cap placement		
		SHO	GWO	GA^20^
Loss cost ($)	150,425.5	59,447	61,120	61,422.22
Peak loss power ($)	55,915.04	21,448	22,782	215,767.29
Capacitor cost ($)	0	24,886	22,411	24,230
Total cost ($)	206,340.54	105,780	106,310	107,228.51
Net saving ($)	–	100,560.54	100,030.54	99,112.03

According to Table 6, spending $ 24,886 annually for SHO's capacitor utilization has led to saving and reducing costs up to approximately 100,560.54 $. The existence of capacitors in the network will reduce losses and improve the voltage profile, and this loss reduction will be accompanied by a reduction in the cost of purchasing power from the upstream network. On the other hand, the cost of installing and maintaining of capacitors compared to a reduction of losses cost is much less. It should also be noted that the lifetime of the capacitors is relatively high and the cost of installing these capacitors will only be in the first year, which could be another reason for the proper operation of this equipment in reducing network costs. To better see the results and performance of the presence of capacitors in the network, the losses amounts, as well as the minimum network voltage before and after the presence of capacitors at different load levels in Tables 7 and 8, are presented in scenarios of connected to the network and islanding mode, respectively. According to Tables 7 and 8, in both scenarios, the presence of capacitors in the network has had a significant positive effect on the reduction of losses, as well as the improvement of the minimum voltage. In Table 8, the performance of the SHO method was compared with the GA method, which confirmed the performance of the proposed method in reducing the losses and improving the minimum voltage of the grid compared to the GA method.

**Table 7:** Losses and minimum load voltage before and after the capacitor placement in the scenario of connected to the network with cosφ = 1.

	SHO			GA^20^		
	Level 1	Level 2	Level 3	Level 1	Level 2	Level 3
**Before cap placement**						
Loss (kW)	46.064	169.018	315.979	46.064	169.018	315.979
Minimum voltage (p.u)	0.9928	0.9779	0.9499	0.9928	0.9779	0.9499
**After cap placement**						
Loss (kW)	28.21	65.40	123.24	28.632	68.311	123.953
Minimum voltage (p.u)	0.9987	0.9916	0.9739	0.9989	0.9894	0.9728

**Table 8:** Losses and minimum load voltage before and after the capacitor placement in the scenario of islanding with cosφ = 1.

	SHO			GA^20^		
	Level 1	Level 2	Level 3	Level 1	Level 2	Level 3
**Before cap placement**						
Loss (kW)	20.670	77.923	149.980	20.670	77.923	149.980
Minimum voltage (p.u)	0.9717	0.9324	0.8892	0.9717	0.9324	0.8892
**After cap placement**						
Loss (kW)	15.00	32.064	57.87	13.641	33.170	55.850
Minimum voltage (p.u)	0.9927	0.9966	0.9716	0.9935	0.9856	0.9605

**Figure 3:**
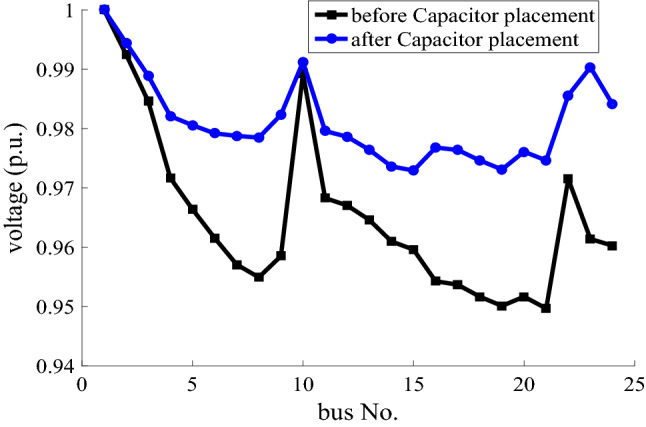
Voltage profile of 24-bus network in the grid-connected mode for the heavy load before and after capacitor placement, with cosφ = 1.

The voltage profiles of the 24-bus network in the grid-connected mode and at the heavy load with cosφ = 1, before and after the capacitor placement are compared in Fig. 3. As it is clear that, the voltage profile is better after the capacitor placement in the network than the base state.

### Simulation results with cosφ#1

**Figure 4:**
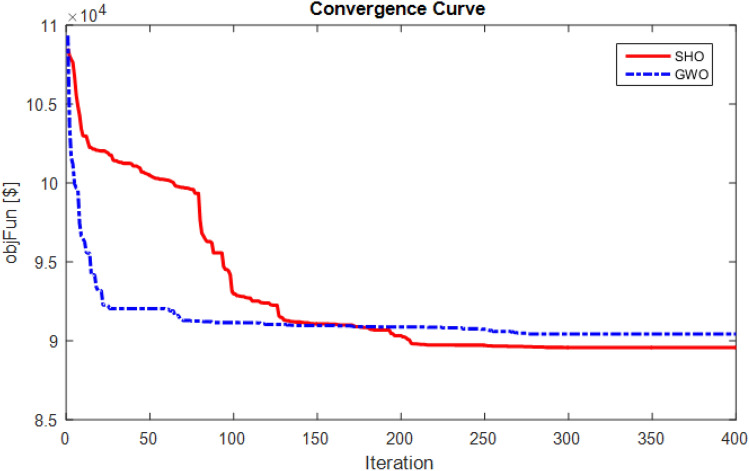
Convergence process optimization methods for the 24-bus network in mode of cosφ#1.

In the second case, it is assumed that distributed generation sources also can generate reactive power and operate under the power factor of 0.95 lag. The convergence curve in this case is also given in Fig. 4, which indicates the achievement of a lower cost value by the SHO method than the GWO method. The value of the cost objective function is obtained by the SHO proposed method equal to 89,568 $ and by the GWO method is 90,423 $ which is obtained by the GA in^45^ equal to 103,098.5$. Therefore, the results indicate that the SHO method is superior to the GWO and GA methods. Also, statistic analysis of different algorithms performance with cosφ#1 due to 25 repetitions is presented in Table 9. According to the obtained results, the SHO is achieved to lower cost in view of Best, Mean, Worst and standard deviation (STD) values compared with the GWO.

**Table 9:** Statistic analysis of different algorithms performance with cosφ#1.

Item/algorithm	SHO	GWO
Best ($)	89,568	90,423
Mean ($)	89,832	90,864
Worst ($)	90,117	91,179
STD ($)	5596	6215

It is clear that due to reactive power injection, in this case, the cost of the capacitor placement is greatly reduced. Also, in this case, the total cost of the system, including the cost of losses and peak losses is severely reduced. Therefore, it can be concluded that the participation of distributed generation resources in the production of reactive power can greatly reduce network costs. The optimal placement of the capacitors in the 24-bus network is presented in Table 10. All three optimization methods have placed the 1650 kV fixed capacitor in the network. At Level 2, the SHO, GWO, and GA^45^ have placed 600, 300 and 1500 kVAR capacitors respectively on the network, and at the third level, have respectively placed, 600, 300 and 2100 kVAR capacitors in the network. In Table 11, the cost of losses, peak losses, cost of capacitor placement, the total cost as well the amount of annual saving is presented. The results show that the cost of losses and cost of peak losses in SHO method decreased more than GA and GWO methods and in total, the annual amount of savings by the SHO was 76,850.46 $. According to Tables 12 and 13, it is observed that in grid-connected and islanded modes the network losses are reduced considerably in different loads using SHO. Also, the network minimum voltage is improved in different loading condition. Also, the SHO method is better in both the network connection scenario and the islanding scenario than the GWO method for reducing losses, as well as the minimum voltage levels at different load levels.

**Table 10:** Optimal capacitor placement at 24-bus network in the mode of cosφ#1.

Bus number	Fixed capacitor			Switchable capacitor (Level 2)			Switchable capacitor (Level 3)		
	SHO	GWO	GA^45^	SHO	GWO	GA^45^	SHO	GWO	GA^45^
7	150	300	150	0	0	0	0	0	0
8	0	0	300	0	0	0	0	0	0
9	0	0	0	150	0	300	150	0	300
11	450	450	300	0	0	0	0	0	0
12	0	0	0	0	150	150	0	150	150
13	0	0	0	150	150	150	150	150	150
14	150	150	300	0	0	0	0	0	0
15	300	150	150	0	0	0	0	0	0
16	0	0	0	150	0	300	150	0	300
17	0	0	0	0	0	300	0	0	300
18	300	300	150	0	0	0	0	0	0
19	0	0	0	150	0	300	150	0	300
20	450	300	300	0	0	0	0	0	0
21	0	0	0	0	0	300	0	0	300
23	0	0	0	0	0	300	0	0	300
Total (kvar)	1650	1650	1650	600	300	1500	600	300	2100

**Table 11:** Different amounts of cost in the network before and after the capacitor placement in the mode of cosφ#1.

Parameter	Before cap placement	After cap placement		
		SHO	GWO	GA^45^
Loss cost ($)	127,389.3	57,099	58,502	62,166.36
Peak loss power ($)	39,029.18	21,448	24,321	20,822.06
Capacitor cost ($)	0	10,192	7599	20,110
Total cost ($)	166,418.48	89,568	90,423	103,098.42
Net saving ($)	–	76,850.46	75,995.48	63,320.06

**Table 12:** Losses and minimum load voltage before and after the capacitor placement in the scenario of connected to the network with cosφ#1.

	SHO			GWO		
	Level 1	Level 2	Level 3	Level 1	Level 2	Level 3
**Before cap placement**						
Loss (kW)	42.158	147.876	232.09	42.158	147.876	232.09
Minimum voltage (p.u)	0.9933	0.9792	0.9589	0.9933	0.9792	0.9589
**After cap placement**						
Loss (kW)	28.873	61.485	125.566	29.277	62.262	134.997
Minimum voltage (p.u)	0.9994	0.9901	0.9724	0.9991	0.9891	0.9695

**Table 13:** Losses and minimum load voltage before and after the capacitor placement in the scenario of islanding at the different load levels with cosφ#1.

	SHO			GA^45^		
	Level 1	Level 2	Level 3	Level 1	Level 2	Level 3
**Before cap placement**						
Loss (kW)	15.996	48.373	92.834	15.996	48.373	92.834
Minimum voltage (p.u)	0.9855	0.9625	0.9249	0.9855	0.9625	0.9249
**After cap placement**						
Loss (kW)	12.930	29.512	60.080	12.975	31.706	67.677
Minimum voltage (p.u)	1.000	0.9882	0.9524	1.000	0.9789	0.9420

The losses and the minimum voltage before and after the capacitor placement in the mode of connected to the network in the second mode at different load levels are given in Table 12.

**Figure 5:**
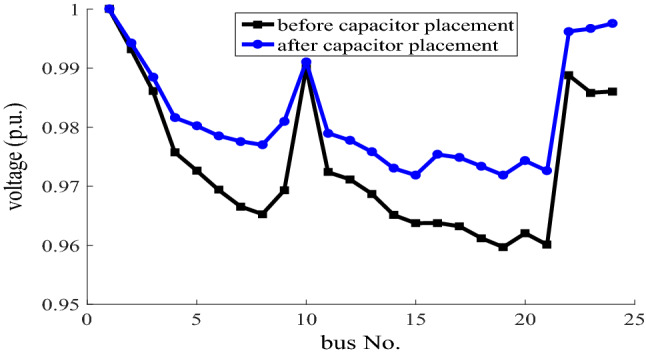
Voltage profile of 24-bus network in the grid-connected mode for the heavy load before and after capacitor placement, with cosφ#1.

The voltage profiles of the 24-bus network in the grid-connected mode and at the heavy load with cosφ#1, before and after the capacitor placement are compared in Fig. 5. As it is clear that, the voltage profile is better after the capacitor placement in the network than the base state.

### Simulation results considering different types of loads

In this section, the effect of different types of loads including constant power, constant current and constant impedance on the cost of losses, cost of energy losses and total annual cost using the SHO method has been studied. In addition to considering the three-level load, different types of load are also included in the problem. Different amounts of cost in a network obtained for different types of loads in the mode of cosφ = 1 and cosφ#1 in Tables 14 and 15 are presented, respectively. According to the results obtained in both cases, the amount of annual cost of the system, the cost of losses of power, energy losses and the cost of the capacitor placement in the constant power load is less than the other types of load. Also by comparing the results in two modes cosφ = 1and cosφ#1, it can be observed that the cost of annual losses, the cost of losses of power, the cost of peak losses, as well as the cost of the capacitor due to the injection of reactive power in the case of cosφ#1 has been reduced. The annual cost reduction in constant power loads, constant current and constant impedance loads was 15.32, 15.92 and 10.79%, respectively.

**Table 14:** Different amounts of cost in the network after the capacitor placement in the mode of cosφ = 1.

Parameter	Constant power	Constant current	Constant impedance
Loss cost ($)	60,520	61,062	61,904
Peak loss power ($)	21,593	22,642	23,380
Capacitor cost ($)	23,150	25,654	22,091
Total cost ($)	105,260	109,360	107,380

**Table 15:** Different amounts of cost in the network after the capacitor placement in the mode of cosφ#1.

Parameter	Constant power	Constant current	Constant impedance
Loss cost ($)	57,095	59,361	60,688
Peak loss power ($)	22,497	23,922	24,928
Capacitor cost ($)	9532	8658	10,177
Total cost ($)	89,124	91,941	95,793

## Conclusion

The optimal allocation of capacitors for distribution network operation is studied in researches to compensate the reactive power. Of course, due to the occurrence of the islanding state in distribution networks, capacitors allocation requires more care and consideration. Therefore, the islanding state of the network can be one of the limitations in the problem solution, which in this study is well addressed and the optimization program must be able to manage the provision of the microgrid load demand in islanding state. In addition, changing of the network load level, as well as observing the voltage range of the buses and the current of the network lines are the other limitations of the study in islanding state. Therefore, using an intelligent and flexible methodology in islanding conditions is critical for problem solution as well as microgrid load supply based on distributed generation sources. In this paper, the optimal allocation of the capacitor for a distribution network in the presence of a microgrid including DG resources is presented using SHO algorithm. Optimal placement of fixed and switchable capacitors is implemented to minimize energy losses, peak losses, and capacitor costs in two grid-connected and islanded modes, considering different load levels. Non-dispatchable distributed generations are considered in two modes: generation and non-generation of reactive power. To solve the optimization problem, the grey wolf algorithm is employed to solve the problem and its implementation on a 24-bus test network. Simulation results are presented in two grid-connected and islanded modes. The simulation results showed that the SHO is a powerful method with high convergence speed for achieving the optimal solution to the problem with the lowest total cost compared to the GWO and GA methods. The total cost value for DGs only with the capability of active power injection is achieved 105,780 $ and 106,310 $ using the SHO and GWO, respectively. Also, this cost for DGs with the capability of active and reactive power injection is obtained 89,568 $ and 90,423 $ using the SHO and GWO, respectively. Moreover, the annual net savings are higher with the SHO method than with the other methods. Furthermore, the results showed that in the case of reactive power injection by distributed generations, the cost of the capacitor placement dropped sharply, and the total cost of the system decreased significantly. Therefore, we conclude that the participation of distributed generations capable of reactive power injection can reduce network costs. The results also cleared that the total cost, loss cost, and peak loss of power in constant power load are less than in the other types of loads. According to the study conducted in this paper, evaluation of power quality indices in islanding conditions is suggested for future work.
